# Successful Nonoperative Management of Small Bowel Obstruction due to Distal Gastric Band Migration: A Rare Late Complication of LAGB

**DOI:** 10.1155/cris/7889399

**Published:** 2026-10-31

**Authors:** Chaled Alnakib, Nuha Riyad, Fahim Kanani, Mohammad Maraqah, Basem Bali, Mordechai Shimonov, Ammir Abuzahra, Osama Ewidat

**Affiliations:** ^1^ Department of Surgery Hebron University Hebron State of Palestine hebron.edu; ^2^ Department of Medicine Faculty of Medicine and Health Sciences An-Najah National University Nablus State of Palestine najah.edu; ^3^ Faculty of Medicine Hebron University Hebron State of Palestine hebron.edu; ^4^ College of Medicine Hebron University Hebron State of Palestine hebron.edu

**Keywords:** bariatric surgery, conservative management, foreign body passage, gastric band migration, laparoscopic adjustable gastric banding, small bowel obstruction

## Abstract

A 61‐year‐old woman presented with abdominal pain, vomiting, and distension 13 years after laparoscopic adjustable gastric banding (LAGB). Imaging showed small bowel obstruction (SBO) caused by distal migration of the gastric band into the jejunum. As she was hemodynamically stable without peritonitis, a conservative management strategy was adopted. Bedside removal of the subcutaneous port was performed to release tubing tension, followed by nasogastric decompression and supportive therapy. Serial imaging confirmed gradual distal movement of the band, which was spontaneously expelled per rectum on the fifth hospital day. The patient recovered fully without surgical intervention. This case highlights the importance of individualized management and demonstrates that conservative therapy, including port removal, can be a safe and effective alternative to surgery for stable patients with distal gastric band migration–related obstruction.

## 1. Introduction

Bariatric surgery remains the most effective long‐term treatment for morbid obesity when conservative approaches fail to achieve sustainable weight loss [1]. Laparoscopic adjustable gastric banding (LAGB), a purely restrictive procedure involving placement of a silicone band around the proximal stomach, gained widespread adoption in the early 2000s due to its technical simplicity, reversibility, and favorable perioperative safety profile [2, 3].

However, LAGB utilization has declined dramatically from 45% of bariatric procedures in 2008 to less than 5.5% currently, primarily due to high long‐term complication rates requiring reoperation in 30%–50% of patients [2, 4]. Major complications include band slippage (2%–14%), port/tubing malfunction (5%–10%), pouch dilation (8%–10%), and band erosion with potential intragastric migration (IGM) (0.3%–11.1%) [5–7].

IGM represents one of the most serious late complications, occurring when chronic mechanical pressure, ischemia, or foreign body reaction causes transmural erosion through the gastric wall [8]. Once intraluminal, peristalsis can propel the band distally, potentially causing mechanical small bowel obstruction (SBO)—a rare but life‐threatening event requiring urgent intervention [9, 10].

Traditional management of migrated bands involves surgical removal via laparoscopy or laparotomy, though these procedures carry significant risks due to adhesions and altered anatomy [11]. Endoscopic retrieval has emerged as an alternative for partially eroded bands accessible within the stomach [12]. Recently, selected case reports have described successful conservative management with spontaneous band expulsion, though no standardized selection criteria or management protocols exist [13, 14].

While the International Federation for the Surgery of Obesity and Metabolic Disorders (IFSO) suggests individualized management may be appropriate in stable patients, the literature lacks consolidated guidance on conservative management of distally migrated bands causing bowel obstruction [15]. Most published cases default to surgical intervention without exploring nonoperative alternatives.

We present a rare case of SBO from distal band migration successfully managed through a conservative approach involving bedside port removal to facilitate spontaneous expulsion. We compare this case with selected published reports to place the management strategy and its limitations in context.

## 2. Case Presentation

A 61‐year‐old woman with a history of LAGB performed in 2008 for morbid obesity presented to the emergency department with a 48‐h history of severe epigastric pain, recurrent vomiting, abdominal distension, constipation, and obstipation, consistent with partial SBO.

Ten years after the initial LAGB placement, the patient began experiencing progressive weight regain associated with upper gastrointestinal symptoms, including dyspepsia and heartburn. An upper endoscopy revealed partial intragastric erosion of the gastric band, indicating band migration through the gastric wall into the lumen. Although endoscopic removal was attempted, it was unsuccessful because of incomplete migration and band fixation. Given the absence of alarming symptoms or complications after the procedure, no further intervention was pursued, and the patient remained under clinical observation. Exact serial weight measurements and a contemporaneous BMI at the current admission were not documented in the material available for this report.

Initial laboratory investigations revealed mild leukocytosis, hypokalemia, hypochloremia, and elevated blood urea nitrogen, consistent with vomiting‐related electrolyte disturbances and mild dehydration. Serum lactate remained within the normal range (Table 1).

**Table 1:** Laboratory parameters on admission demonstrating mild dehydration and stress response.

Test	Result	Unit	Normal range	Clinical significance
White blood cell count	**11.8**	×10^3^/μL	4.0–11.0	Mild leukocytosis (stress response)
Hemoglobin	13.2	g/dL	12.0–16.0	Normal
Platelet count	285	×10^3^/μL	150–450	Normal
Sodium	138	mmol/L	135–145	Normal
Potassium	**3.3**	mmol/L	3.5–5.0	Mild hypokalemia (vomiting)
Chloride	**96**	mmol/L	98–106	Mild hypochloremia (vomiting)
Bicarbonate	28	mmol/L	22–28	Mild metabolic alkalosis
Creatinine	0.9	mg/dL	0.6–1.2	Normal
BUN	**24**	mg/dL	7–20	Mild elevation (dehydration)
C‐reactive protein	**8.2**	mg/L	<5	Mild elevation
Lactate	1.2	mmol/L	0.5–2.0	Normal
Albumin	3.8	g/dL	3.5–5.0	Normal

*Note:* Comprehensive metabolic and hematological assessment performed at emergency department presentation, ~48 h after symptom onset. Values reflect physiological response to partial bowel obstruction with vomiting, including mild hypochloremic, hypokalemic metabolic alkalosis, and prerenal azotemia. Notably absent are signs of systemic infection (normal WBC, mild CRP elevation only) or tissue ischemia (normal lactate), supporting the decision for conservative management. All values are normalized by hospital day 3 following fluid resuscitation and nasogastric decompression. Abnormal values are highlighted in bold.

Plain abdominal radiography showed the subcutaneous gastric‐band port in its original subxiphoid position (Figure 1). Axial contrast‐enhanced CT confirmed the subcutaneous port within the anterior abdominal wall (Figure 2). Contrast‐enhanced CT localized the band to the distal duodenum/proximal jejunum (Figure 3), with marked upstream gastric (Figure 4) and duodenal dilatation (Figure 5) and distal bowel decompression, supporting a transition point and partial mechanical SBO rather than isolated gastric dilatation. The silicone connecting tube remained intact and extended from the migrated band to the subcutaneous port (Figure 6).

**Figure 1:**
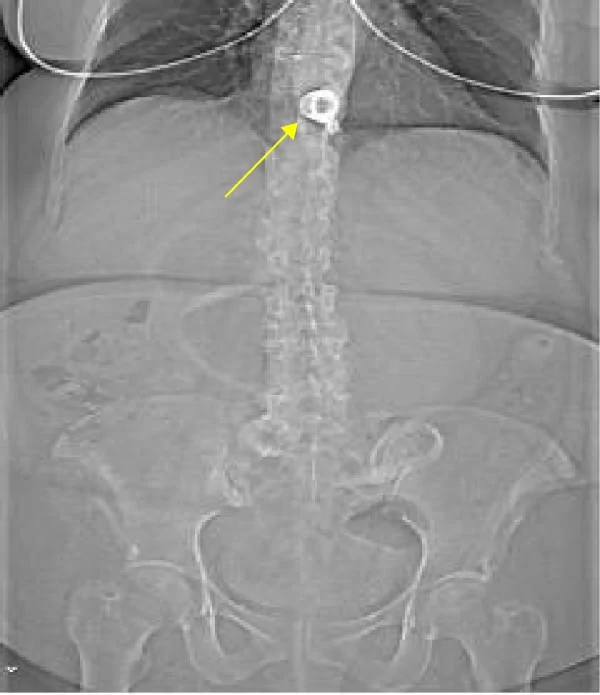
Initial abdominal radiograph demonstrating intact LAGB system components. Plain anteroposterior abdominal radiograph obtained on admission showing the radiopaque subcutaneous access port (yellow arrow) in its expected left subxiphoid position. The metallic port appears well‐positioned without evidence of displacement or rotation. No gastric band is visible at the expected gastroesophageal junction location, suggesting complete intragastric migration. Multiple air‐fluid levels are present throughout the small bowel, consistent with partial obstruction.

**Figure 2:**
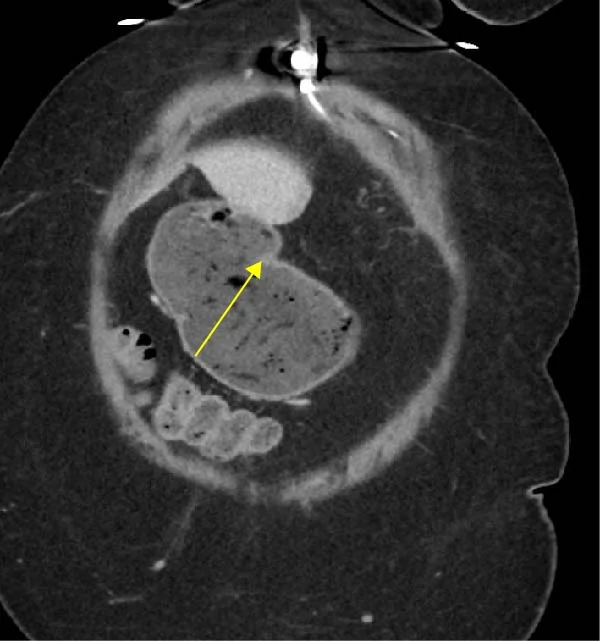
Axial contrast‐enhanced CT confirming subcutaneous port position. Axial CT image at the level of the upper abdomen demonstrates the intact subcutaneous access port (yellow arrow) embedded within the anterior abdominal wall soft tissues. No surrounding fluid collection or soft tissue stranding is identified, excluding port site infection. The silicone connecting tube extends from the port toward the peritoneal cavity (not shown in this slice).

**Figure 3:**
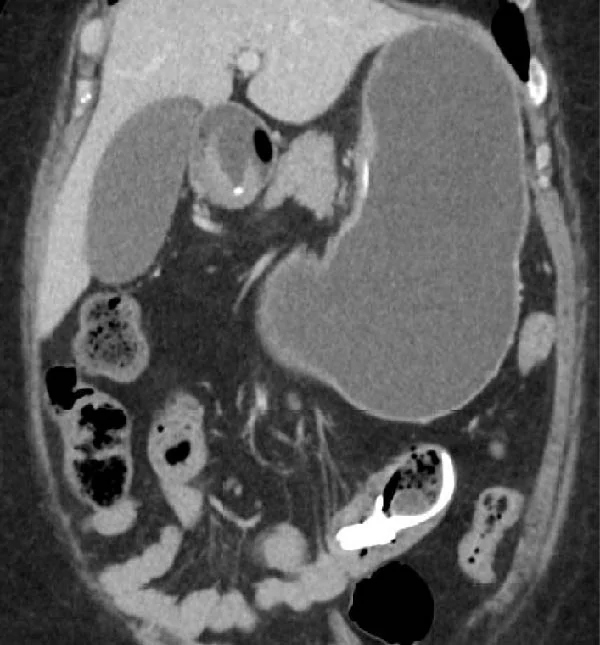
Coronal CT localizing migrated band within small bowel. The band appears intact and circular without evidence of fragmentation. Proximal bowel dilatation and distal decompression confirm the site of partial mechanical obstruction. This favorable position allowed for conservative management.

**Figure 4:**
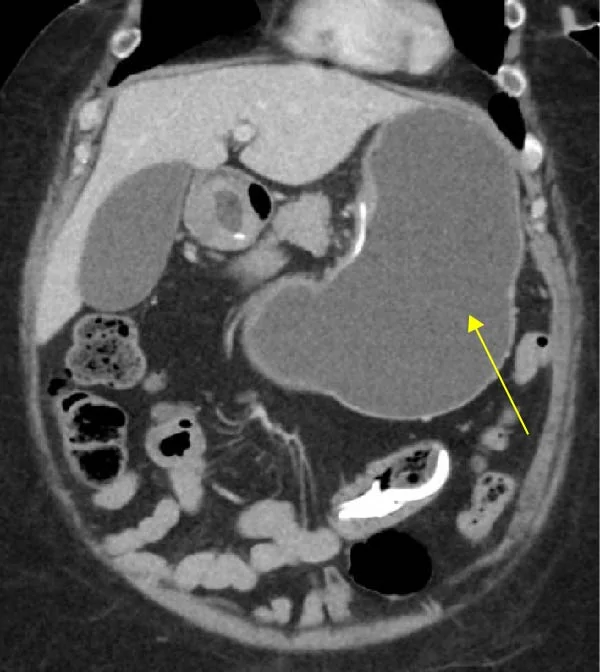
Coronal CT demonstrating severe gastric distention. The stomach appears grossly dilated with smooth wall contours and no evidence of pneumatosis. This finding suggests functional gastric outlet obstruction secondary to the migrated band. Note the decompressed distal stomach (arrow).

**Figure 5:**
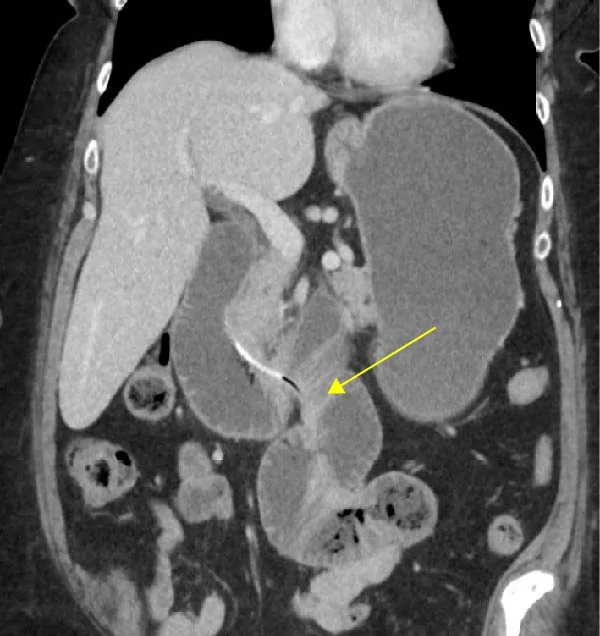
Coronal CT revealing duodenal obstruction pattern. The transition point suggests obstruction at the duodenojejunal junction. Air‐fluid levels in the proximal small bowel indicate partial mechanical obstruction. The dilated duodenum forms a characteristic “C‐loop” configuration.

**Figure 6:**
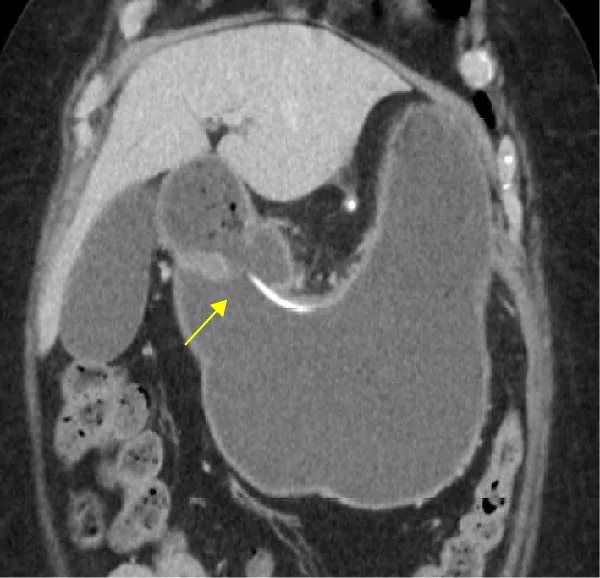
Sagittal CT showing intact LAGB tubing course. The tube remains continuous without evidence of disconnection or kinking. The dilated stomach is visible posteriorly. This intact connection likely contributed to the band’s tethering within the bowel lumen.

Given the absence of hemodynamic instability or peritoneal signs and considering the anatomical complexity of the duodenal region, the decision was made to pursue conservative management. After multidisciplinary discussions involving bariatric surgeons, radiologists, and gastroenterologists, the patient underwent bedside removal of the subcutaneous port under local anesthesia. A 1.5 cm transverse incision was made in the subxiphoid region under sterile conditions, and ~5 mL of 1% lidocaine was infiltrated subcutaneously. The port was carefully dissected and disconnected from the silicone tube, which was then released to migrate freely within the bowel lumen (Figure 7).

**Figure 7:**
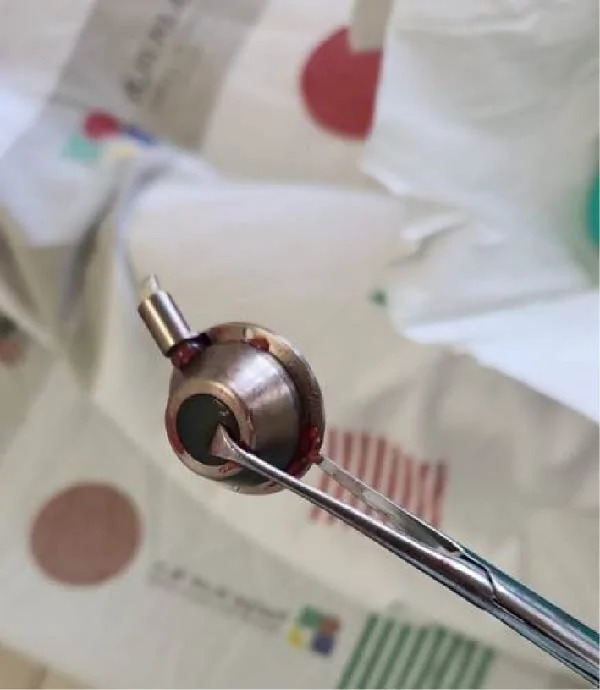
Extracted subcutaneous port following bedside removal. Intraoperative photograph showing the successfully removed LAGB subcutaneous access port held in surgical forceps. The metallic port measures ~2.5 cm in diameter with the silicone connecting tube still attached. The tube was intentionally transected to allow free intraluminal migration of the gastric band. No evidence of port infection or degradation is present.

The patient was monitored clinically three times per day during the first 2 days to detect any signs of deterioration, such as fever, hypotension, pain, or laboratory changes warranting surgical conversion. During this period, the NGT output decreased progressively: initially draining 2 L of bilious “pea soup” content, which later became clearer and less in quantity. She began passing flatus, and the NGT was subsequently removed.

She was then managed expectantly with intravenous hydration, electrolyte correction, stool softeners, and close clinical and radiological monitoring to track the band’s progression through the GI tract (Figure 8).

**Figure 8:**
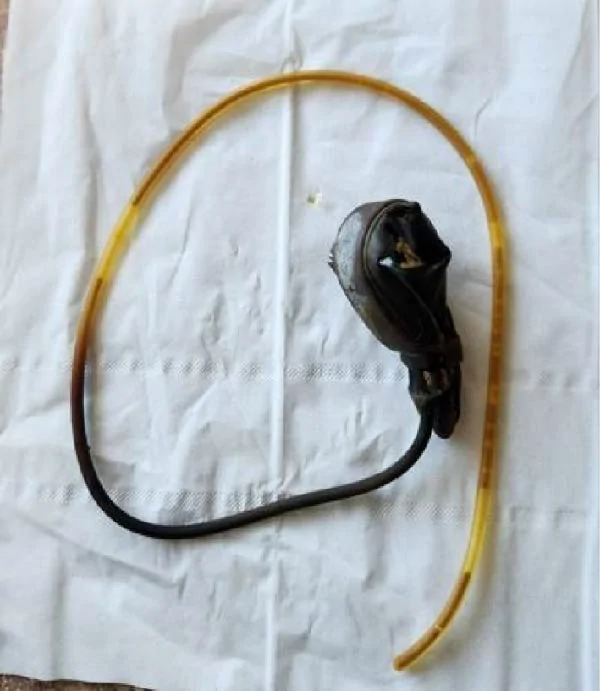
Expelled gastric band after successful conservative management. Photograph of the intact laparoscopic adjustable gastric band following spontaneous per‐rectal passage on hospital day 5. The silicone band measures ~5 cm in diameter with attached tubing segment measuring 8 cm. The band remains completely intact without fragmentation or sharp edges, confirming the safety of its transintestinal passage. This represents successful nonoperative management of a potentially life‐threatening complication.

Over the next 5 days, the patient remained stable with no signs of bowel perforation or worsening obstruction. On day 5 of hospitalization, the gastric band was spontaneously expelled in the stool without complications (Figure 9). Follow‐up abdominal imaging confirmed complete transit and the absence of retained components.

**Figure 9:**
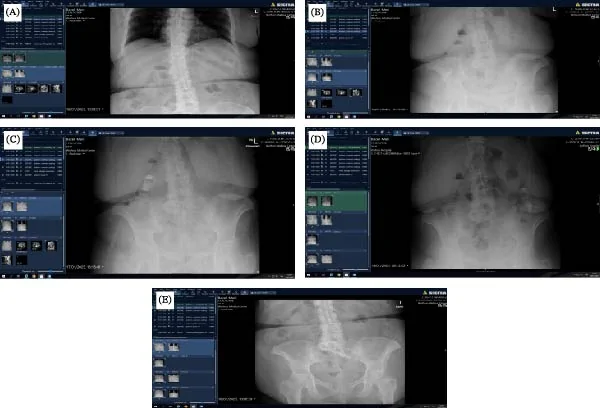
Serial abdominal radiographs documenting spontaneous band passage. Sequential anteroposterior abdominal radiographs obtained over 5 days showing progressive distal migration of the gastric band through the gastrointestinal tract. (A) (Day 0): Band visible in right upper quadrant (proximal jejunum). (B) (Day 1): Migration to mid‐abdomen (mid‐jejunum). (C) (Day 2): Band in right lower quadrant (ileum). (D) (Day 4): Passage into left colon. (E) (Day 5): Band in rectosigmoid region before expulsion progressive resolution of small bowel dilatation is evident throughout the series.

Following expulsion, the patient’s symptoms resolved completely. She tolerated oral intake and was discharged home in stable condition with instructions for follow‐up.

## 3. Discussion

### 3.1. Pathophysiology

Gastric band erosion and migration result from chronic mechanical pressure inducing localized ischemia and transmural necrosis of the gastric wall. Foreign body reaction and subclinical infections accelerate this process, with biofilm formation contributing to persistent inflammation [1, 6]. Once intraluminal erosion occurs, peristalsis propels the band distally through the gastrointestinal tract.

In our case, the intact connecting tube likely served as a “rail,” guiding migration through the pylorus into the small bowel. This mechanism differs from fragmented band migration, where disconnected components move unpredictably [6]. The 5‐year interval between documented erosion and obstructive presentation aligns with literature reports of prolonged asymptomatic periods before clinical manifestation.

The pathophysiology explains why many patients remain stable despite an intraluminal foreign body—gradual erosion rather than acute perforation allows time for local adaptation and inflammation resolution. Understanding this mechanism informed our decision that immediate surgery might not be mandatory if clinical parameters remained favorable. The intact band configuration and proximal jejunal location suggested potential for safe passage through the remaining bowel, provided that mechanical constraints were addressed.

### 3.2. Management Rationale

Our decision for conservative management balanced risks against potential benefits. Surgical extraction of migrated bands carries significant morbidity due to adhesions and altered anatomy [1, 5]. Endoscopic removal, while less invasive, has limited success for bands beyond the duodenal sweep [3, 10]. Both approaches risk complications including perforation, bleeding, and anastomotic leak.

The key innovation was bedside port removal under local anesthesia. This simple intervention eliminated the tethering effect of the connecting tube, transforming a fixed obstruction into a free‐floating object capable of spontaneous passage. By releasing mechanical constraints while maintaining close monitoring, we facilitated natural expulsion while preserving surgical options if deterioration occurred.

Our monitoring protocol emphasized the early detection of complications through systematic assessment:

• Clinical examination every 8 h
• Daily laboratory markers
• Serial abdominal radiographs tracking band progression
• Clear criteria for surgical conversion

The decreasing nasogastric output and return of bowel function by day 3 provided objective evidence of resolving obstruction. We maintained a low threshold for intervention while allowing the body’s natural mechanisms to work.

The conservative plan was continued only while the patient remained clinically stable and showed objective improvement in NGT output, bowel function, laboratory findings, and serial radiographs. This approach avoided extensive operative dissection while preserving surgery as an immediate rescue option. These conditions were reviewed jointly by the surgery, radiology, and gastroenterology teams.

### 3.3. Literature Comparison

Published reports show that surgical or endoscopic retrieval remains common [1, 8, 10]. Martines et al. reported operative retrieval for migrated LAGB [1], while Sleiman et al. [8] described endoscopic removal of an eroded band that had migrated into the proximal jejunum. Bains et al. [16] reported a 35‐year‐old woman with weight regain and recurrent port‐site discharge after LAGB; endoscopy confirmed IGM, but retrieval was unsuccessful, and dense operative adhesions later necessitated conversion from laparoscopy to open gastrotomy for band removal. In contrast, our patient had distal migration into the proximal jejunum with partial SBO; after the subcutaneous port was removed and tubing tension was released, the intact band progressed distally and was passed spontaneously. These cases share late erosion/migration and weight regain as indicators of band failure but differ in band location, tethering, and the feasibility of nonoperative passage. Selected published cases are summarized in Table 2.

**Table 2:** Selected published cases of gastric band erosion or migration: management approaches and outcomes.

Study	Band location	Intervention	Outcome	Notes
Sleiman et al. [8]	Proximal jejunum	Endoscopic removal	Successful	Eroded band
Lemaire et al. [12]	Jejunum	Spontaneous passage	Successful	Conservative approach
Kim et al. [10]	Distal stomach	Endoscopic	Successful	Partial migration
Bains et al. [16]	Intragastric	Laparoscopic converted to open removal	Successful	Weight regain; recurrent port‐site discharge; dense adhesions
Current case	Proximal jejunum	Port removal **+ ** conservative	Spontaneous passage	Novel mechanism (tube detachment)

*Note:* Selected published reports are summarized for clinical context; no systematic search or pooled analysis was performed. The table compares band location, intervention, outcome, and key clinical features relevant to the present case.

Our case therefore differs from both extraction‐based management and passive observation: Port removal was used as a limited intervention to release the tethering effect of the tube before expectant spontaneous passage.

Because the available evidence is limited to case reports, conservative management should be considered only in highly selected patients. Features favoring observation in the present case included clinical stability without sepsis or peritonitis, partial rather than complete obstruction, an intact band without sharp or fragmented components, continued distal progression on imaging, and immediate access to operative rescue.

The foreign body literature provides supportive evidence. Conservative management has succeeded with various ingested objects when specific criteria are met [13–15]. The ASGE guidelines classify smooth, blunt, and radiopaque objects as lower risk, potentially suitable for expectant management [17]. Gastric bands fit this category when intact and appropriately positioned.

Unlike passive observation, bedside port removal actively modified the mechanical constraint by releasing the tethering effect of the connecting tube while preserving the option of surgical rescue.

The 5‐day interval to spontaneous expulsion was accompanied by progressive clinical and radiographic improvement. Although the uncomplicated transit in this patient is encouraging, evidence is limited to case reports, and this single outcome cannot establish definitive selection criteria.

### 3.4. Clinical Implications

This case does not support routine nonoperative treatment of migrated bands. In this patient, the strategy was considered because the obstruction was partial, the patient was hemodynamically stable without peritoneal signs, multidisciplinary serial monitoring was feasible, and immediate operative rescue remained available.

Continued observation requires repeated reassessment with clinical examination, laboratory testing, NGT output and bowel‐function monitoring, and serial imaging. Complete obstruction, perforation, peritonitis, sepsis, hemodynamic instability, failure of distal progression, or loss of objective improvement should prompt operative management.

Because evidence for this strategy consists primarily of case reports, the present outcome should be interpreted cautiously. Additional reports are needed before formal selection criteria or a standardized protocol can be proposed; conservative management should remain a closely monitored option for selected stable patients rather than a substitute for timely surgery when deterioration occurs.

## Author Contributions

Chaled Alnakib and Nuha Riyad (shared first authorship) conceived and designed the study, collected clinical data, analyzed results, and drafted the manuscript. Fahim Kanani, Mohammad Maraqah, and Osama Ewidat contributed to data collection, literature review, and manuscript editing. Basem Bali contributed to endocrinological evaluation, data interpretation, and manuscript preparation. Mordechai Shimonov and Osama Ewidat are senior authors, supervised the study, provided critical revisions, and approved the final manuscript version. Ammir Abuzahra contributed to the conceptualization of the revised manuscript, validation, writing – review and editing, and project administration. Ammir Abuzahra contributed to the interpretation of the clinical and literature findings, approved the final version, and agrees to be accountable for the integrity of the work.

## Funding

This research received no specific grant or financial support from any funding agency in the public, commercial, or not‐for‐profit sectors.

## Disclosure

All authors have read and approved the final version of the manuscript.

## Ethics Statement

The study was conducted in accordance with the Declaration of Helsinki. Documentation is available upon request. This study was approved by the Wolfson Medical Center Institutional Review Board (Approval Number WMC‐0156‐23) with a waiver of informed consent due to its retrospective design. All procedures were conducted in accordance with the Declaration of Helsinki and institutional guidelines.

## Consent

No identifiable patient data were included, and therefore, consent for publication was not required. Written informed consent to publish the case and images was obtained from the patient.

## Conflicts of Interest

The authors declare no conflicts of interest.

## Supporting Information

Additional supporting information can be found online in the Supporting Information section.

## Supporting information


**Supporting Information** The CARE checklist for case reports has been completed and is provided as Supporting File 2 to ensure comprehensive and transparent reporting of this case.

## Data Availability

Data are available upon requests from the authors.

## References

[1] Martines G. , Picciariello A. , Ugenti I. , Lagovardou E. , Digennaro R. , and Capuano P. , Laparoscopic Adjustable Gastric Banding Migration: An Early Approach for a Late Complication, Giornale di Chirurgia - Journal of Surgery. (2017) 38, no. 5, 225–228, 10.11138/gchir/2017.38.5.225. PMC5761634 29280701

[2] Vitiello A. , Berardi G. , Velotti N. , De Palma G. D. , and Musella M. , Is There an Indication Left for Gastric Band? a Single Center Experience on 178 Patients With a Follow-Up of 10 years, Updates in Surgery. (2021) 73, no. 2, 657–662, 10.1007/s13304-020-00858-8. PMC8005389 32737822

[3] Collado-Pacheco D. , Rábago-Torre L. R. , and Arias-Rivera M. , et al.Endoscopic Extraction of Adjustable Gastric Bands After Intragastric Migration as a Complication of Bariatric Surgery: Technique and Advice, Endoscopy International Open. (2016) 04, no. 6, E673–E677, 10.1055/s-0042-105868. PMC4993909 27556077

[4] Alawad M. , Abukhater M. , and Al-Mohaimeed K. , Eroded Adjustable Gastric Band Migration Causing Gastric Obstruction and Perforation in a Pregnant Lady, International Journal of Surgery Case Reports. (2020) 71, 192–195, 10.1016/j.ijscr.2020.04.087. PMC7533630 32473550

[5] Nguyen M. , Aulick S. , and Walters T. , Catheter Displacement and Penetration of a Vaginal Cuff Following Laparoscopic Adjustable Gastric Banding: A Case Report, Cureus. (2024) 16, no. 8, 10.7759/cureus.67497. PMC11416128 39310413

[6] Aili A. , Li X. , and Abudureyimu K. , Intra-Jejunal Migration With Intestinal Obstruction and Perforation After Gastric Banding: A Case Report, Heliyon. (2023) 9, no. 10, 10.1016/j.heliyon.2023.e20756. PMC10582381 37860574

[7] Didziokaite G. , Poskus T. , Malakauskiene L. , and Ramasauskaite D. , Penetration of the Gastric Band Through the Gastric Wall During Pregnancy: A Rare Case Report, International Journal of Surgery Case Reports. (2021) 89, no. C, 10.1016/j.ijscr.2021.106640, 106640. PMC8645911 34864267

[8] Sleiman A. , Studer A. S. , and Garneau P. Y. , et al.Case Report: Endoscopic Removal of an Eroded Gastric Band Causing Small Bowel Obstruction upon Migration Into the Proximal Jejunum, Obesity Surgery. (2020) 30, no. 12, 5153–5156. 10.1007/s11695-020-04906-5 PMC7417257 32779076

[9] Hota P. , Caroline D. , Gupta S. , and Agosto O. , Laparoscopic Adjustable Gastric Band Erosion With Intragastric Band Migration: A Rare but Serious Complication, Radiology Case Reports. (2018) 13, no. 1, 76–80, 10.1016/j.radcr.2017.11.012. PMC5826467 29487641

[10] Kim S. Y. , Oh K. Y. , and Chung J. W. , et al.Endoscopic Treatment of Diverse Complications Caused by Laparoscopic Adjustable Gastric Banding: A Study in Eastern Asia, Gut and Liver. (2017) 11, no. 4, 497–503, 10.5009/gnl16089. PMC5491084 28335099

[11] Eisenberg D. , Shikora S. A. , and Aarts E. , et al.American Society of Metabolic and Bariatric Surgery (ASMBS) and International Federation for the Surgery of Obesity and Metabolic Disorders (IFSO) Indications for Metabolic and Bariatric Surgery, Obesity Surgery. (2023) 33, no. 1, 3–14, 10.1007/s11695-022-06332-1. PMC9834364 36336720

[12] Lemaire J. , Dewit O. , and Navez B. , Management of a Jejunal Obstruction Caused by the Migration of a Laparoscopic Adjustable Gastric Banding. A Case Report, International Journal of Surgery Case Reports. (2017) 30, no. C, 6–8, 10.1016/j.ijscr.2016.11.026. PMC5129149 27898356

[13] Husain R. , Alghamdi D. A. , Ghzoi F. A. , AlArafah S. K. , Bahammam M. A. , and Al Duhileb M. , Conservative Management of Fish Bone-Induced Large Bowel Perforation: Case Report, International Journal of Surgery Case Reports. (2022) 95, no. C, 10.1016/j.ijscr.2022.107157, 107157. PMC9112106 35569310

[14] Molla Y. D. , Gelaw T. B. , Alemu H. T. , Kassie M. T. , and Sisay M. A. , Spontaneous Passage of Ingested Sewing Needle: A Case Report, Clinical Case Reports. (2024) 12, no. 5, 10.1002/ccr3.8857. PMC11077285 38721550

[15] Pokharel S. , Kc S. , and Lamichhane S. , et al.Asymptomatic Spontaneous Expulsion of a Long Foreign Body Through the Gastrointestinal Tract – a Curious Case Report, Radiology Case Reports. (2024) 19, no. 5, 1940–1944, 10.1016/j.radcr.2024.02.007. PMC10915782 38449486

[16] Bains L. , Lal P. , Vindal A. , and Gautam K. K. , Weight Regain After LABG: Ponder to Intra-Gastric Migration of Adjustable Gastric Band, MAMC Journal of Medical Sciences. (2020) 6, no. 2, 139–142, 10.4103/mamcjms.mamcjms_78_19.

[17] Birk M. , Bauerfeind P. , and Deprez P. , et al.Removal of Foreign Bodies in the Upper Gastrointestinal Tract in Adults: European Society of Gastrointestinal Endoscopy (ESGE) Clinical Guideline, Endoscopy. (2016) 48, no. 5, 489–496, 10.1055/s-0042-100456. 26862844

