# Supplementary material for: Successful Nonoperative Management of Small Bowel Obstruction due to Distal Gastric Band Migration: A Rare Late Complication of LAGB
Source: Case Rep Surg. 2026 Oct 31;2026:7889399. doi: 10.1155/cris/7889399 (PMC13723311; doi:10.1155/cris/7889399)
Supplement: Supplementary file 1 [file CRIS-2026-7889399-s001.pdf]

CARE Checklist of information to include when writing a case report

CARE case report guidelines

(cc) BY-NC-ND

Topic	Item	Checklist item description	Reported on Line
Title	1	The diagnosis or intervention of primary focus followed by the words "case report"	Title page
Key Words	2	2 to 5 key words that identify diagnoses or interventions in this case report, including "case report"	Abstract (Keywords Section)
Abstract (no references)	3a	Introduction: What is unique about this case and what does it add to the scientific literature?	Abstract, first sentence
	3b	Main symptoms and/or important clinical findings	Abstract, middle part
	3c	The main diagnoses, therapeutic interventions, and outcomes	Abstract, last sentences
	3d	Conclusion—What is the main "take-away" lesson(s) from this case?	Abstract, final sentence
Introduction	4	One or two paragraphs summarizing why this case is unique (**may include references**)	Introduction section, paragraphs 1
Patient Information	5a	De-identified patient specific information.	Case presentation
	5b	Primary concerns and symptoms of the patient.	Case presentation, first paragraph
	5c	Medical, family, and psycho-social history including relevant genetic information	Case presentation, past medical histo
	5d	Relevant past interventions with outcomes	Case presentation, prior endoscopic attempt
Clinical Findings	6	Describe significant physical examination (PE) and important clinical findings.	Case presentation, physical exam
Timeline	7	Historical and current information from this episode of care organized as a timeline	Case presentation and Fig. 8 radiographs timeline
Diagnostic Assessment	8a	Diagnostic testing (such as PE, laboratory testing, imaging, surveys).	Case presentation, CT and labs (Table 2)
	8b	Diagnostic challenges (such as access to testing, financial, or cultural)	Discussion, management rationale
	8c	Diagnosis (including other diagnoses considered)	Case presentation
	8d	Prognosis (such as staging in oncology) where applicable	Discussion, clinical implicatioı
Therapeutic Intervention	9a	Types of therapeutic intervention (such as pharmacologic, surgical, preventive, self-care)	Case presentation, port removal & conservative therapy
	9b	Administration of therapeutic intervention (such as dosage, strength, duration)	Case presentation, bedside procedure details
	9c	Changes in therapeutic intervention (with rationale)	Case presentation, NG tube removal, supportive care
Follow-up and Outcomes	10a	Clinician and patient-assessed outcomes (if available)	Case presentation, outcome paragraph
	10b	Important follow-up diagnostic and other test results	Case presentation, serial imaging and radiographs
	10c	Intervention adherence and tolerability (How was this assessed?)	Case presentation, well-tolerated
	10d	Adverse and unanticipated events	None reported
Discussion	11a	A scientific discussion of the strengths AND limitations associated with this case report	Discussion, pathophysiology & management rationale
	11b	Discussion of the relevant medical literature **with references**.	Discussion, literature comparison
	11c	The scientific rationale for any conclusions (including assessment of possible causes)	Discussion, clinical implications
	11d	The primary "take-away" lessons of this case report (without references) in a one paragraph conclusion	Discussion, final paragraph
Patient Perspective	12	The patient should share their perspective in one to two paragraphs on the treatment(s) they received	Discussion, final paragraph
Informed Consent	13	Did the patient give informed consent? Please provide if requested	Yes ☒ No ☐
